# Supplementary figures and images for: Quorum sensing regulators and non-ribosomal peptide synthetases govern antibacterial secretions in Xenorhabdus szentirmaii
Source: Front Microbiol. 2025 Mar 12;16:1560663. doi: 10.3389/fmicb.2025.1560663 (PMC11936946; doi:10.3389/fmicb.2025.1560663)

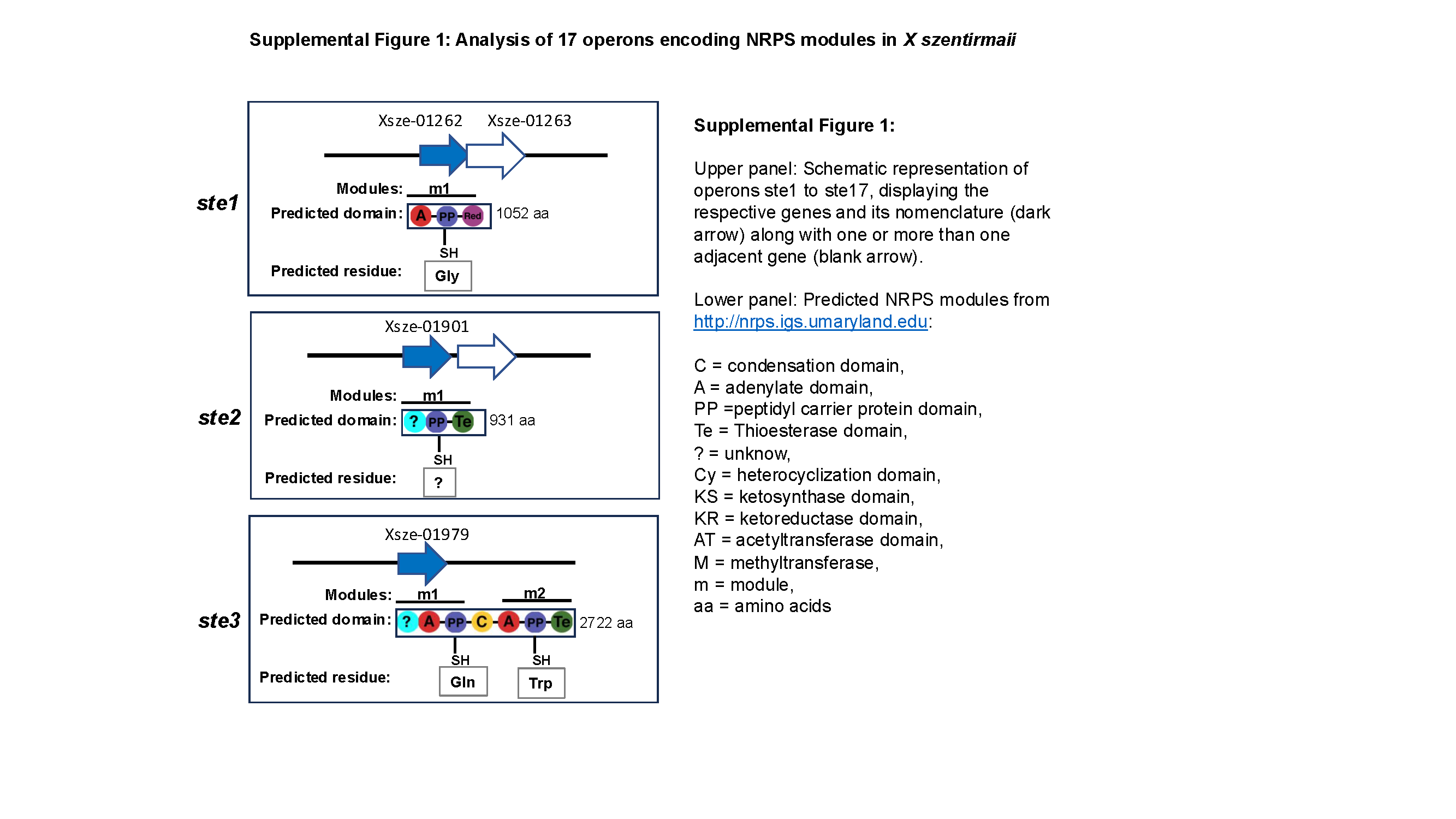

Supplement: Supplementary file 1 [file Image_1.TIFF]

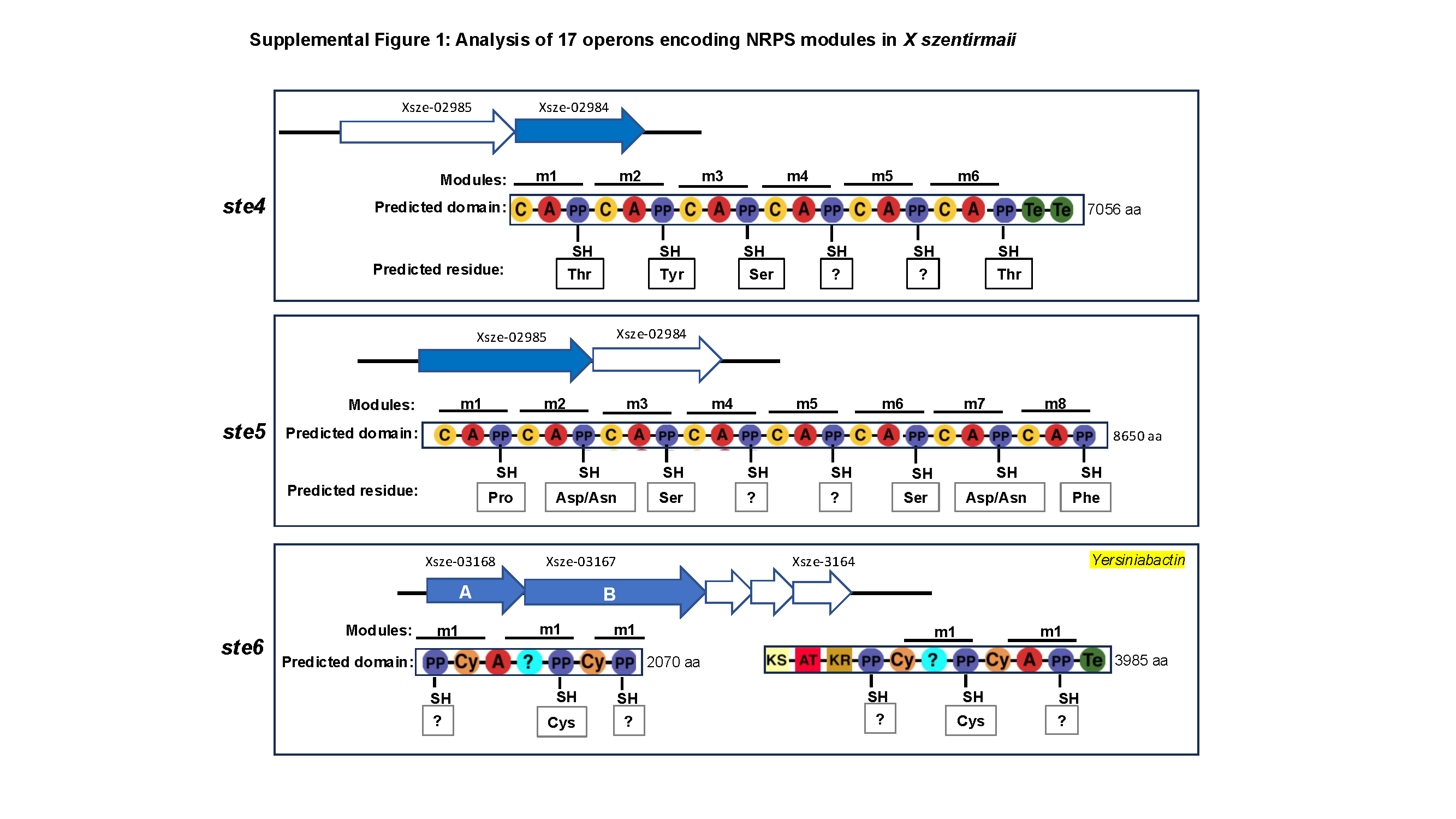

Supplement: Supplementary file 2 [file Image_2.TIFF]

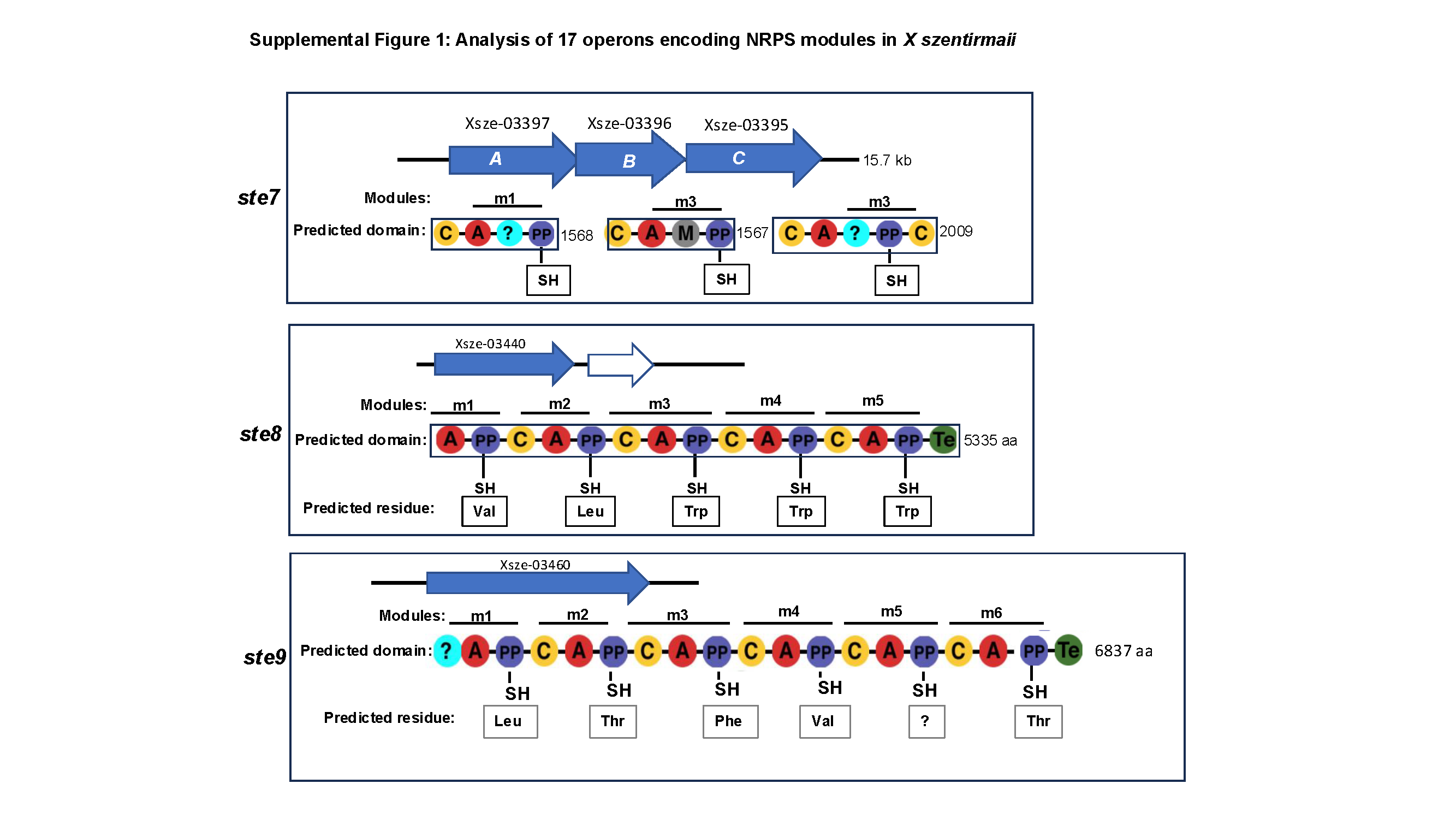

Supplement: Supplementary file 3 [file Image_3.TIFF]

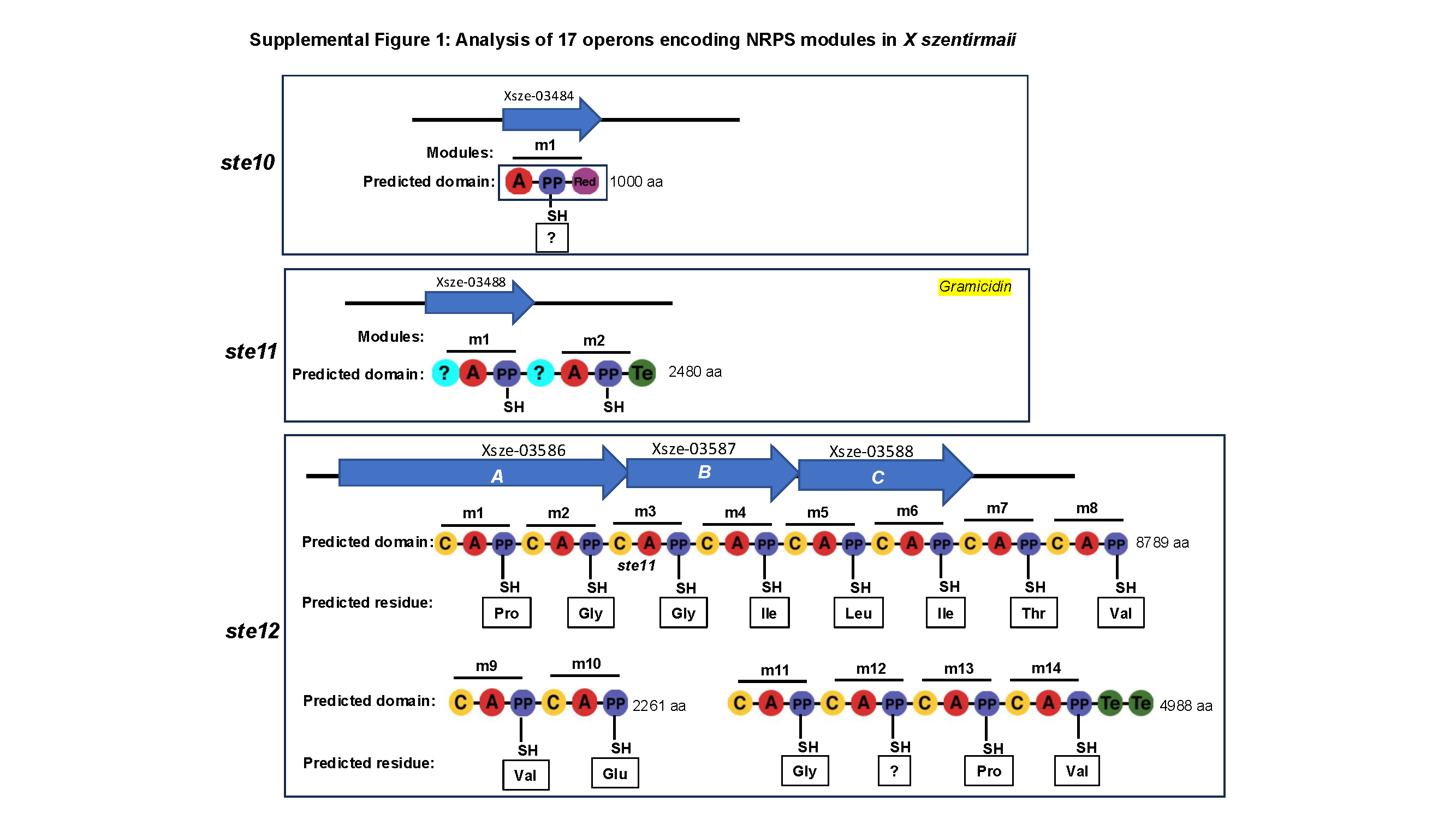

Supplement: Supplementary file 4 [file Image_4.TIFF]

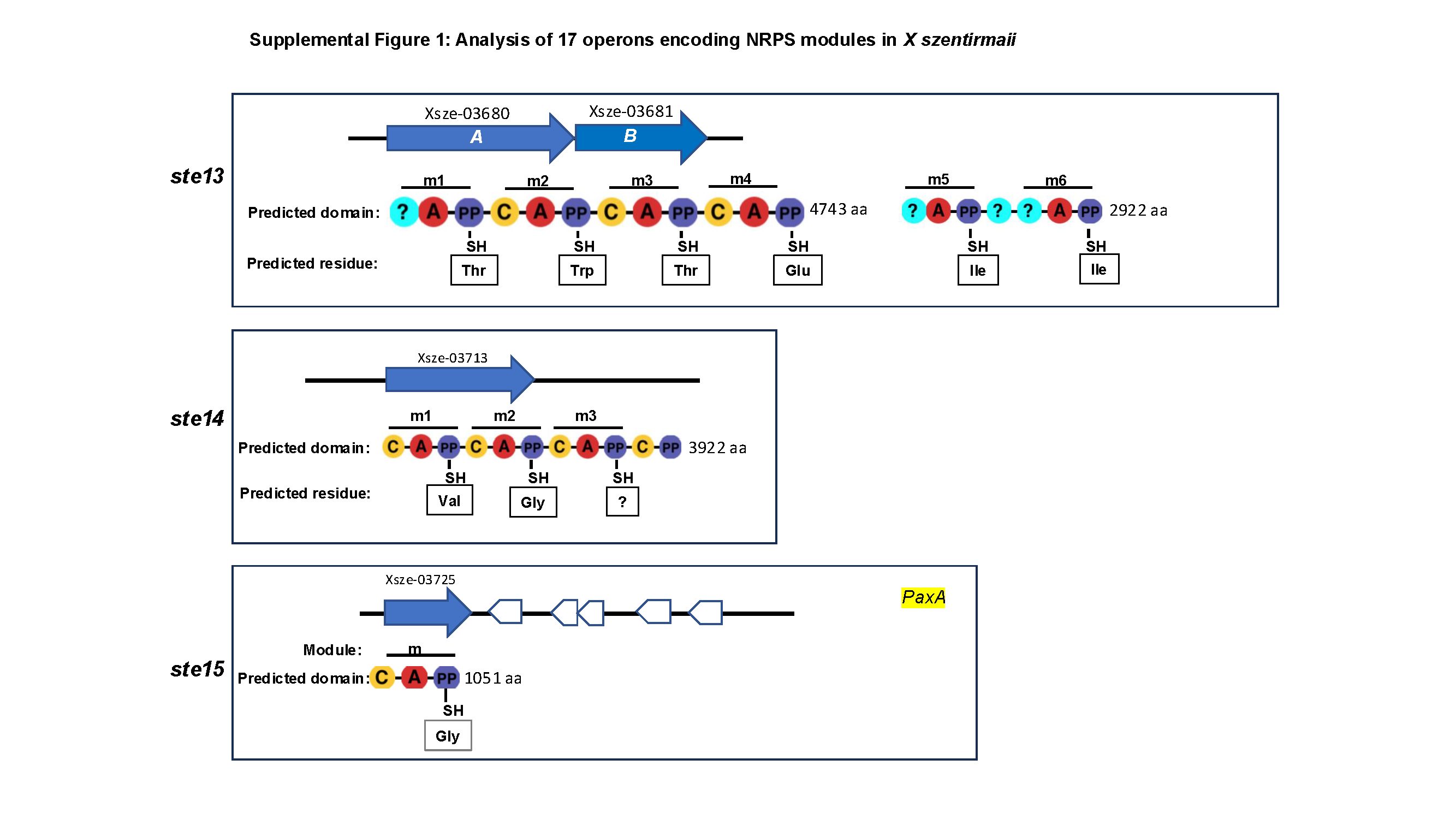

Supplement: Supplementary file 5 [file Image_5.TIFF]

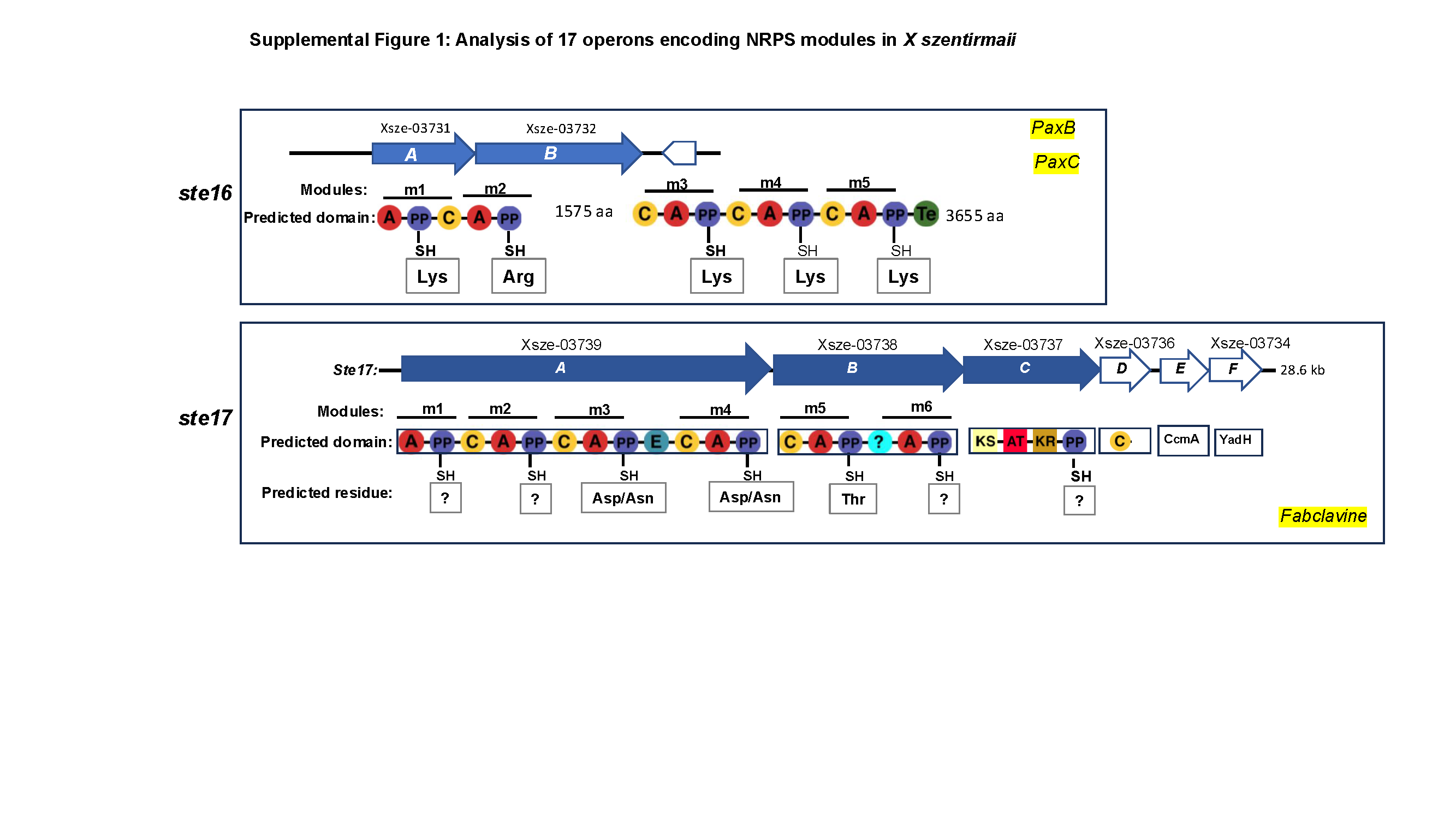

Supplement: Supplementary file 6 [file Image_6.TIFF]

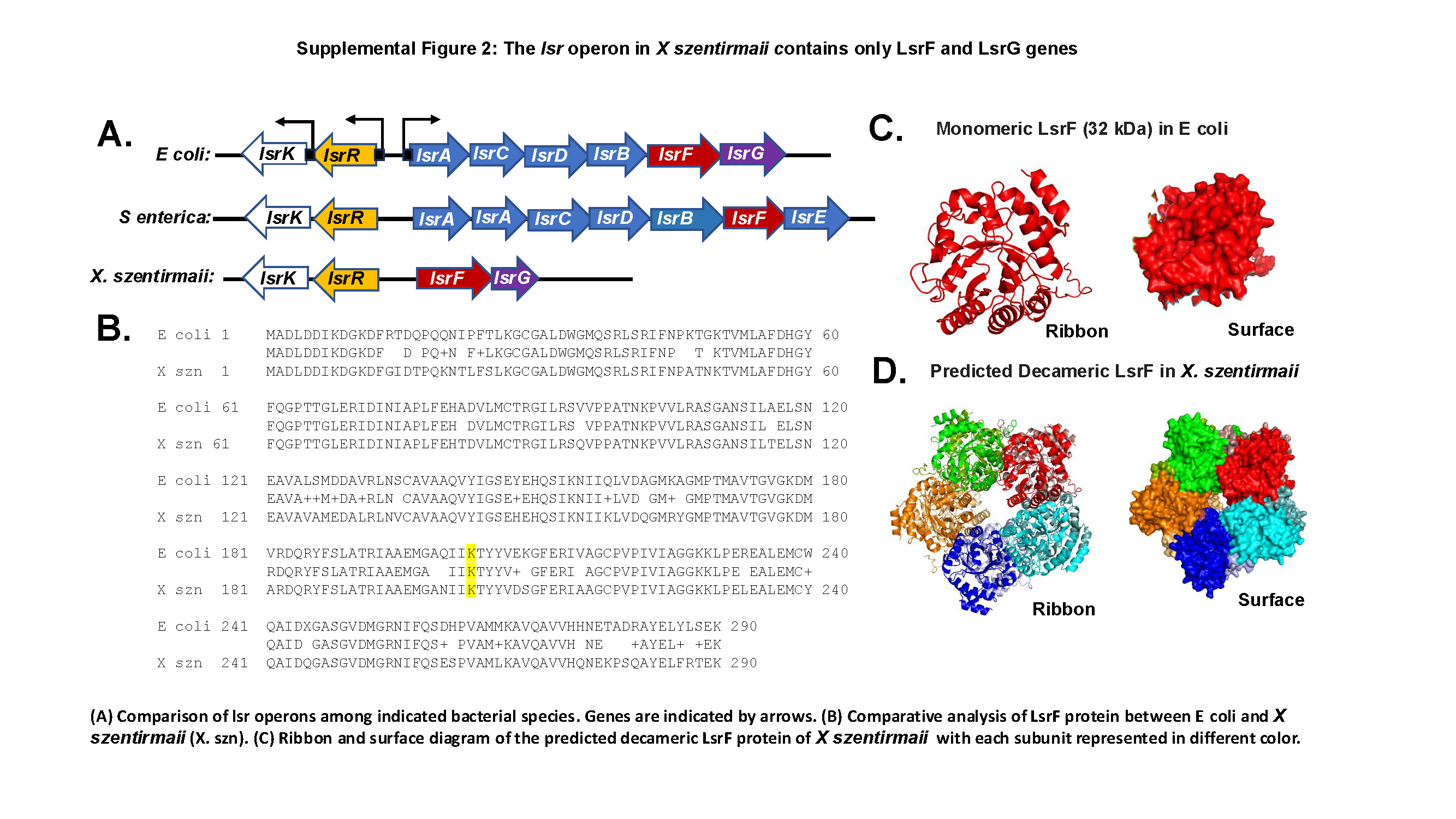

Supplement: Supplementary file 7 [file Image_7.TIFF]
